# An Ultra-Compact ARCL-Based MEMS Radar Filter for Mobile Robotic Platforms

**DOI:** 10.3390/mi17070830

**Published:** 2026-07-11

**Authors:** Yan Ding, Ruiqi Zhang, Xing Fan, Wenyu Chen, Zhe Yang

**Affiliations:** 1 School of Information and Control Engineering, University of Emergency Management, Langfang 101601, China; zhangruiqi@ncist.edu.cn (R.Z.); fanguan123@126.com (X.F.); 17526712912@163.com (W.C.); 2 Key Laboratory of Brain-Computer Interface Technology Application of the Ministry of Emergency Management, Langfang 101601, China; 3 Nanjing Electronic Devices Institute, Nanjing 210002, China; zheyang_nedi@163.com

**Keywords:** ARCL, MEMS, filter, miniaturization, low loss

## Abstract

To address the stringent requirements for miniaturization and high reliability in the perception systems of mobile robotic platforms, this article presents an ultra-compact bandpass filter based on air core recta-coax lines using micro-electro-mechanical systems technology. The proposed filter features an air-filled cavity structure with internal coupled lines and a fully enclosed metal shield, which effectively minimizes dielectric and radiation losses while achieving a highly compact footprint. This compactness is particularly critical for robotic radar front-ends, where limited payload capacity demands high integration density. By leveraging classical filter synthesis theory, the design achieves a high-order response within a minimized volume. Furthermore, the inherent high-Q characteristic of the air cavity significantly improves out-of-band rejection, thereby effectively suppressing interference in complex electromagnetic environments and enhancing the signal-to-noise ratio for robotic detection. A prototype operating at 75 GHz was fabricated and measured. The experimental results demonstrate a low insertion loss of 1.5 dB and a compact size of 0.875 mm^3^, showing reasonable agreement with simulations. The proposed design offers a promising solution for next-generation, high-performance sensing units in autonomous robotics.

## 1. Introduction

Mobile robotic platforms, such as autonomous mobile robots (AMRs) and surgical assistive robots, increasingly rely on millimeter-wave radar sensors to achieve reliable perception in densely cluttered and dynamically changing environments [1,2]. To meet the stringent size, weight, and power constraints inherent to these platforms, the front-end modules of such radar systems demand core filtering components that simultaneously offer ultra-low insertion loss, high selectivity, extreme miniaturization, and robust interference suppression [3]. Among these, the bandpass filter (BPF), as a critical circuit block, directly determines the signal-to-noise ratio (SNR) and anti-interference capability of the entire radar receiver [4].

Conventional BPF implementations have been extensively explored using planar printed circuit boards (PCB), low-temperature co-fired ceramics (LTCC), substrate-integrated waveguides (SIW), metallic waveguide resonators, and semiconductor-based integrated passive devices (IPDs) [5,6,7,8,9,10]. However, at millimeter-wave frequencies, each of these technologies exhibits inherent limitations. PCB and LTCC architectures suffer from substantial dielectric losses, which severely degrade insertion loss and radar detection range [5,6]. SIW structures, while offering better radiation suppression, still incur non-negligible dielectric and conductor losses; moreover, their miniaturization via half-mode or quarter-mode techniques compromises electromagnetic shielding and introduces additional radiation loss [7,8]. Metallic waveguide resonators provide high quality factors and power handling but at the cost of a prohibitively large footprint, which is incompatible with the limited payload capacity of mobile robots [9]. Conversely, semiconductor-based IPDs achieve high integration density but fundamentally sacrifice power handling and insertion loss performance due to lossy silicon substrates [10]. Consequently, novel filter architectures must be investigated to concurrently achieve miniaturization, low loss, and high selectivity—requirements that are increasingly critical for next-generation robotic radar front-ends.

Air-filled, fully enclosed metal cavities, implemented using high-precision micro-electro-mechanical systems (MEMS) technology, present a compelling alternative. By leveraging an air-gapped cavity and a continuous metal shield, these structures virtually eliminate dielectric loss and radiation loss, while maintaining high power handling capacity and broadband operation [11,12,13]. Among these, the air core recta-coax line (ARCL) has recently demonstrated significant advantages in low-loss phase shifters [14], broadband antenna arrays [15], and compact power splitters [16]. To date, several ARCL-based BPFs have been reported, primarily employing branch-line topologies. Specifically, the resonant unit in [17] consists of a metal cavity, a rectangular metal block suspended inside, and a grounded cylinder. The filter in [18] utilizes grounded and open stub lines, together with impedance transformation stub lines. The design in [19] adopts half-wavelength resonators and realizes coupling between resonant units by adding grounded cylinders. These works have fully exploited the advantages of ARCL, achieving low insertion loss and compact form factors. However, in the context of extreme miniaturization for robotic radar applications, it remains worth investigating whether more suitable topologies can be adopted based on the ARCL platform to achieve further size reduction without compromising electrical performance.

In response to the above challenges, this article proposes an ultra-compact ARCL-based MEMS bandpass filter specifically tailored for mobile robotic radar platforms. Unlike previous designs that rely on grounding cylinders or stub structures for inter-resonator coupling, the proposed filter adopts a parallel-coupled line configuration, where coupling is achieved through lateral gaps. This topological choice offers a more compact footprint and more precise coupling control. By applying classical filter synthesis theory, a high-order passband response is achieved while maintaining a minimized physical volume. The air-filled, metal-enclosed structure inherently provides ultra-low insertion loss of 1.5 dB and significantly improved out-of-band rejection, thereby enhancing the radar’s ability to suppress interference in spectrally congested environments. A prototype operating at 75 GHz is fabricated using MEMS technology and measured. Experimental results show excellent agreement with simulations. The proposed design offers a promising path toward high-performance, compact, and interference-resilient perception modules for autonomous mobile robotics.

## 2. Design and Simulation

### 2.1. Filter Configuration

**Figure 1:**
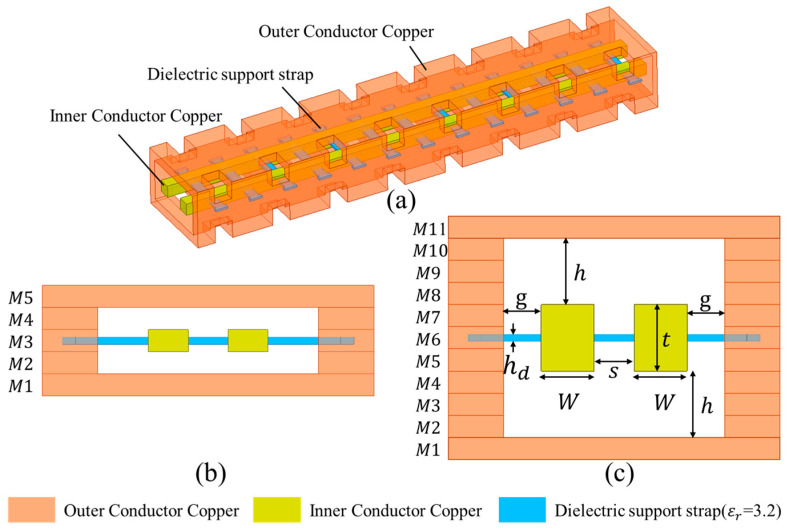
(**a**) 3D view, (**b**) half-height, and (**c**) full-height cross-sectional view of defined coupled ARCL.

Figure 1a presents the 3D view of the proposed coupled ARCLs, comprising the outer conductor copper, the inner conductor copper, and the embedded dielectric support straps. Figure 1b,c illustrate the corresponding cross-sectional views of the transmission line structures fabricated using different lamination schemes. The structure is realized through a multilayer process where each metal layer has a thickness of 50 μm. As shown in Figure 1b, the structure spans from M1 to M5, with the inner conductor co-fabricated within the M3 layer. In contrast, Figure 1c depicts a structure extending from M1 to M11, where the inner conductor is integrated with layers M5–M7. It is worth noting that the thickness and vertical position of the inner conductor are flexible, which can be synchronously fabricated with other layer combinations, such as M6, M4–M8, or M3–M9, depending on the design requirements. While increasing the number of layers enhances the degrees of freedom for realizing complex high-performance devices, it inevitably increases the fabrication complexity. Furthermore, the release holes in the outer conductor are sized at 300 μm×250 μm×100 μm with a periodic arrangement of 750 μm. Similarly, the dielectric support straps have a width of 150 μm and a period of 750 μm. These dimensions are optimized to ensure sufficient mechanical strength while effectively facilitating the removal of the sacrificial photoresist during the release process.

To analyze the characteristics of a single signal line, the spacing s between the coupled lines is set to zero. This configuration effectively merges the two parallel conductors into a single equivalent conductor, and the structure operates as a standard ARCL. Prior to the analysis of coupled lines, we first investigate the characteristics of this single-conductor configuration. The characteristic impedance $Z_{0}$ is determined by [20]:

(1) $$Z_{0}=\frac{1}{v_{p}C_{len}}$$

where $v_{p}\approx 2.998\times 10^{8}m/s$ is the phase velocity in the air medium, and $C_{len}$ denotes the capacitance per unit length. For the physical dimensions defined in the cross-section where 2W≥g, t≥h and 2W≥t, $C_{len}$ can be analytically calculated following the formulation in [21]:

(2) $$C_{len}=2\varepsilon (\frac{2W}{h}+\frac{t}{g})+\frac{4\varepsilon }{\pi }[ln(\frac{g^{2}+h^{2}}{{4h}^{2}})+2\frac{h}{g}\arctan\left(\frac{g}{h}\right)]+\frac{4\varepsilon }{\pi }[ln(\frac{g^{2}+h^{2}}{{4g}^{2}})+2\frac{g}{h}\arctan\left(\frac{h}{g}\right)]$$

when t<h, $C_{len}$ is determined by [21]:

(3) $$C_{len}=2\frac{2\varepsilon W}{h}+\frac{4\varepsilon }{\pi \ln\left(2\right)}ln[1+coth(\frac{\pi g}{2h+t})]\cdot [\frac{t+2h}{2h}ln(\frac{4h+t}{t})+ln(\frac{t\left(4h+t\right)}{{4h}^{2}})]$$

**Figure 2:**
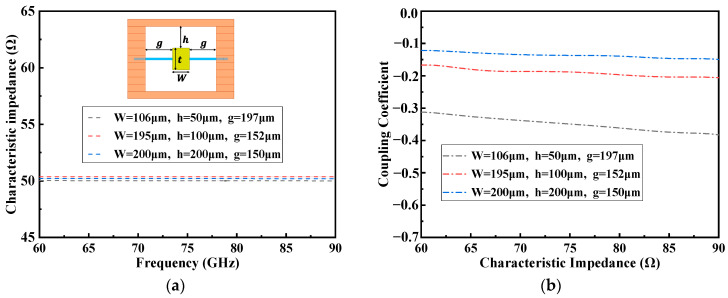
(**a**) Three cross-sectional dimension combinations under 50 Ω characteristic impedance, and (**b**) the ideal insertion loss of ARCL with a length of 1 cm for the three cross-sectional dimensions.

While the physical dimensions are initially synthesized using analytical equations, precise extraction and verification are performed using Ansys HFSS 2021R1. To achieve a characteristic impedance $Z_{0}$ of 50 Ω within the target frequency band, the geometric dimensions of the ARCLs were optimized. As illustrated in Figure 2a, three distinct cross-sectional configurations were investigated by varying the center conductor height h to  50 μm, 100 μm, and 200 μm. For each height, the conductor width W and gap g were tuned to maintain impedance matching. The analytical calculations yielded impedances of 49.9 Ω, 50.3 Ω, and 50.1 Ω, respectively, showing excellent agreement with the full-wave simulation results presented in Figure 2a. Figure 2b compares the ideal insertion loss for a 1 cm transmission line length across these three configurations. A significant reduction in insertion loss is observed as the center conductor height increases. Specifically, at 75 GHz, the structure with h=50 μm exhibits an insertion loss approximately 2.3 times that of the h=200 μm configuration. This degradation in loss performance for thinner conductors is primarily attributed to the increased conductor loss. A smaller cross-sectional height intensifies current density and exacerbates the skin effect, thereby raising the effective resistance. However, realizing a thicker center conductor inevitably entails a more complex fabrication process, involving additional lamination or plating steps. Consequently, a trade-off between electrical performance and manufacturing complexity is required. For the experimental validation in this work, the most fabrication-friendly 5-layer structure (M1–M5) with a fixed unit layer thickness of 50 μm was selected. By strictly controlling the width W and gap g, this study aims to verify the design methodology and process reliability. Once validated, this design framework can be extended to thicker, low-loss configurations for applications requiring ultra-low insertion loss.

Building upon the validated single-line model, we extend the topology to coupled transmission lines by introducing a second inner conductor. The proximity of the two conductors alters the electric field distribution, thereby modifying the equivalent capacitance per unit length. To achieve a 50 Ω matched coupled line, we adopt the width derived from the single-line analysis as the initial design value. Subsequently, EM optimization is performed to fine-tune both the width W and the spacing s to compensate for the coupling effects and ensure impedance matching. To evaluate the impedance and coupling coefficient of the coupled ARCL, finite-element method simulations are carried out at 75 GHz. The line length is initially set to a quarter of the guided wavelength to maximize the coupling coefficient.

The physical length of the ARCL $l_{ARCL}$ corresponding to a full guided wavelength at the desired center frequency $f_{0}$ can be estimated by:

(4) $$l_{ARCL}=\frac{c}{f_{0}}$$

where c is the speed of light in a vacuum. It should be noted that the actual physical wavelength in the fabricated ARCL will be slightly shorter than the value calculated by the equation above, due to the parasitic capacitance introduced by the dielectric support straps and the finite thickness of the conductors.

For a transmission line with a port impedance of $Z_{0}$, the odd-mode and even-mode impedances, $Z_{0o}$ and $Z_{0e}$, satisfy the well-known relations [22]:

(5) $$Z_{0e}Z_{0o}=Z_{0}^{2}$$

The coupling coefficient can be expressed in terms of $Z_{0o}$ and $Z_{0e}$, as follows:

(6) $$K=\frac{Z_{0e}-Z_{0o}}{Z_{0e}+Z_{0o}}$$

**Figure 3:**
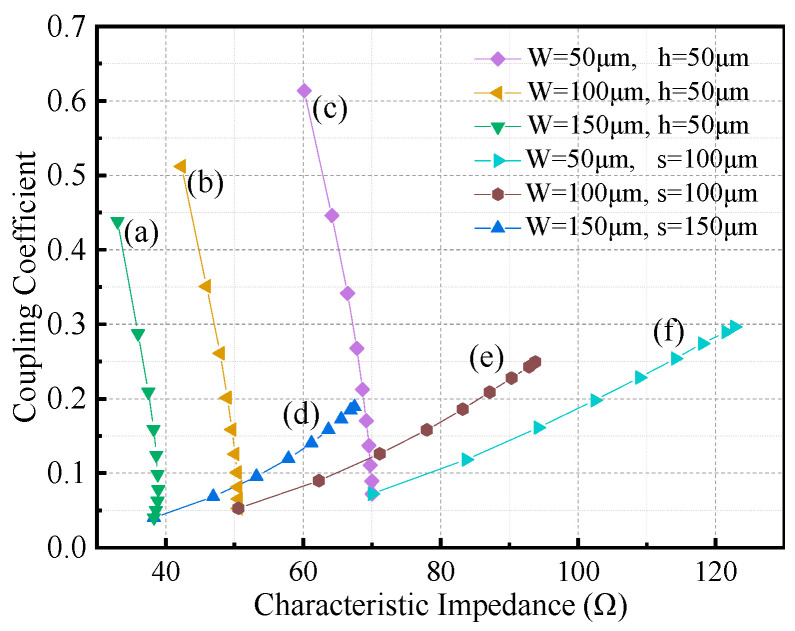
Coupling coefficient versus characteristic impedance for coupled ARCLs. (a–c) Variations in characteristic impedance with gap s for fixed W and h: (a) W=150 μm, h=50 μm (b) W=100 μm, h=50 μm; (c) W=50 μm, h=50 μm. (d–f) variations in characteristic impedance with height h for fixed W and s: (d) W=150 μm, s=150 μm; (e) W=100 μm, s=100 μm; (f) W=50 μm, s=100 μm.

FEM simulation is employed to extract $Z_{0o}$ and $Z_{0e}$, from which the relationship between the coupling coefficient and the characteristic impedance of the coupled ARCL structure is derived, as shown in Figure 3. With the conductor width W and height h fixed as defined in Figure 1c, reducing the coupling gap s enhances the electromagnetic interaction between the signal lines, thereby increasing the coupling coefficient, as shown in curves (a)–(c). When W and s are constant, increasing h alters the equivalent capacitance and inductance distribution. As observed in curves (d)–(f), a larger h leads to an increase in characteristic impedance, accompanied by a rising trend in the coupling coefficient. These characteristic curves provide a basis for the initial synthesis of broadband filters. For a given system impedance and target coupling strength, a smaller gap s is preferred, whereas h can be adjusted without significantly degrading the coupling performance. As the filter in this work is designed with both h and t fixed to 50 μm, the curves in Figure 3(a)–(c) are adopted for selecting the impedance and coupling coefficient.

### 2.2. Filter Design, Tuning, and Analysis

**Figure 4:**
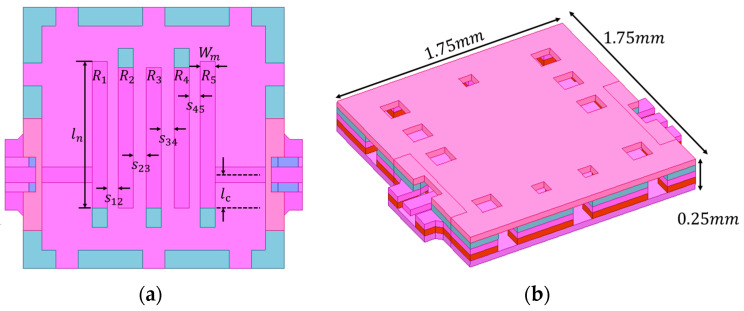
Configuration of the proposed W-band fifth-order MEMS cavity filter: (**a**) Key dimensions of the center conductor, and (**b**) 3D view.

In this work, a fifth-order resonant circuit operating at W-band ($f_{0}=75\;GHz$) is designed. It achieves a 10 GHz bandwidth and a return loss greater than 15 dB, while maintaining sufficient out-of-band rejection. As shown in Figure 4a, the circuit consists of five resonator units ($R_{1},R_{2},R_{3},R_{4},R_{5}$) arranged in a staggered parallel configuration. The resonators are characterized by widths $W_{m}$ (m=1,2,…,5) and lengths $l_{n}$ (n=1,2,…,5), with the gap between adjacent resonators denoted as $s_{ij}$ (i =1, 2, …, 4; j = i + 1). The mutual coupling strength between adjacent resonators is governed by their geometric parameters, particularly the widths and spacing. The prototype filter coefficients ${\;g}_{i}$ (i = 0, 2, …, 6) are derived from standard low-pass prototype tables [22] as $g_{0}{\;=\;g}_{6}\;=\;1$, $g_{1}{\;=\;g}_{5}\;=\;1.1468$, $g_{2}{\;=\;g}_{4}\;=\;1.3712$, $g_{3}\;=\;1.975$. The coupling coefficients $K_{ij}$ and the external quality factor $Q_{e}$ at the input are derived by the following equations:

(7) $$k_{ij}=\frac{BW}{f_{c}\sqrt{g_{i}g_{i+1}}}$$

(8) $$Q_{e}=\frac{g_{0}g_{1}}{BW}f_{c}$$

Based on the target design specifications, the coupling coefficients and external quality factor are calculated using Equations (7) and (8), yielding $K_{12}=0.11$, $K_{23}=0.08$, and $Q_{e}=8.6$. Due to the symmetry of the topology, the corresponding values $K_{34}$, $K_{45}$ and the external quality factor at the output port are also obtained. The design of the first two resonators is initiated by targeting a coupling coefficient $K_{12}=0.11$. As illustrated in Figure 3(a)–(c), for a fixed width W and height h, a coupling of 0.11 is satisfied at characteristic impedances of 39 Ω and 52 Ω. The results further indicate that for an inner conductor width W ranging from 50 μm to 100 μm, appropriate coupling gaps can be found to achieve the desired coupling. Balancing fabrication complexity, conductor loss, and impedance matching with the feed line, the initial dimensions for these resonators are selected as a width of 100 μm and a coupling gap of 65 μm. Based on these initial values, full-wave simulations are performed to refine the resonator dimensions for the target resonant frequency.

Figure 4 shows the configuration of the proposed W-band fifth-order MEMS cavity filter. Figure 4a illustrates the key dimensions of the center conductor, where the dashed lines indicate the feed length $l_{c}$ at the input and output ports. According to the topology, each resonator unit is open-circuited at one end and grounded at the other. The open end is realized by maintaining sufficient horizontal and vertical distance from the outer conductor, while the ground connection is established by stacking the center conductor M3 with metal layers M2 and M4, and directly connecting to M1 and M5. Figure 4b presents a 3D view of the filter. To enable robust on-chip characterization and facilitate integration with external planar circuits, ground-signal-ground (GSG) probe pads designed in previous work [23] are adopted at both the input and output ports.

**Figure 5:**
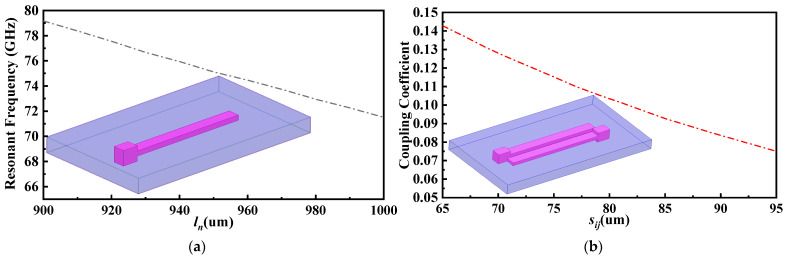
(**a**) Variation in the resonant frequency as a function of $l_{0}$ (black solid line), and (**b**) coupling coefficient $K_{ij}$ as a function of the coupling gap $s_{ij}$ (red dashed line). The magenta solid model boxes represent the inner conductors, and the dark blue regions represent the outer conductor walls with ideal conductor boundaries.

To extract the resonant frequency, a simulation model consisting of a quarter-wavelength metal conductor and an air cavity is established for a single resonator, as shown in Figure 5a. The outer walls of the air cavity are set as finite good conductors, serving as the inner walls of the outer conductor. Here, $l_{0}$ and $W_{0}$ denote the length and width of the resonator, with initial values set to 1000 μm and 100 μm, respectively. Figure 5a plots the variation in the resonant frequency as a function of $l_{0}$. As $l_{0}$ increases, the equivalent capacitance rises, leading to a decrease in the resonant frequency. Specifically, a length of approximately 952 μm yields a resonant frequency of 75 GHz, which aligns well with the target specification.

Based on the optimized length of the single resonator, a two-resonator model was constructed with its initial spacing determined from the curves in Figure 3. Figure 5b plots the simulated coupling coefficient $K_{ij}$ as a function of the coupling gap $s_{ij}$, from which the required $s_{ij}$ can be obtained. From the simulated curve in Figure 5b, the initial coupling gaps corresponding to the target coefficients $K_{12}=0.11$ and $K_{23}=0.08$ are read as approximately $s_{12}=76\;\mu m$ and $s_{23}=91\;\mu m$, respectively.

With the dimensions of the first two resonators determined, the design proceeds to the remaining resonator units. Owing to the structural symmetry of the fifth-order filter topology, the coupling coefficients satisfy $K_{34}=K_{23}$ and $K_{45}=K_{12}$. Consequently, the geometric parameters for the third, fourth, and fifth resonators are directly derived from those already obtained for the first two resonators, following the same design procedure. Once all resonators are dimensioned, the input and output tap-feed structures are designed. The tap position and stub length $l_{c}$ annotated in Figure 4 are optimized by matching the simulated group delay τ to its theoretical value derived from the external quality factor $Q_{e}$. The group delay at resonance is given by [24]:

(9) $$Q_{e}=\frac{\omega_{0}\tau (\omega_{0})}{4}$$

where $\omega_{0}$ is the angular frequency at the center frequency, and $\tau \left(\omega_{0}\right)$ denotes the group delay at the center frequency. Using the designed $Q_{e}$ value, the target group delay at the center frequency is calculated to be 73 ps. A parametric simulation is then performed to evaluate the relationship between the feed length $l_{c}$ and the resulting group delay τ. Based on the simulation, a feed length $l_{c}$ of 225 μm is found to yield a group delay closest to the analytical prediction of 73 ps and is therefore adopted as the optimal feed geometry. By symmetry, the same tap dimensions are applied to the output port. The complete filter model is then assembled and verified through full-wave simulation.

**Figure 6:**
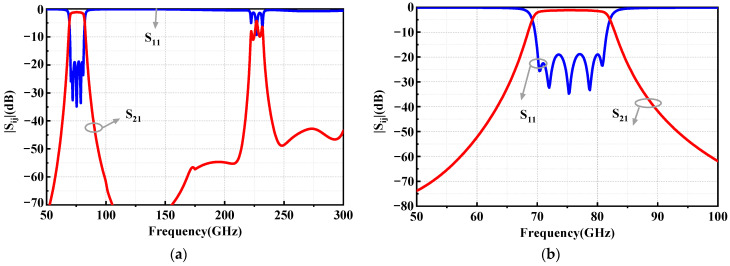
Simulated performance of the optimized filter model (**a**) 50–300 GHz, and (**b**) 50–100 GHz. The blue and red lines represent $S_{11}$ and $S_{21}$, respectively.

The simulated S-parameters of the optimized filter are presented in Figure 6. Figure 6a illustrates the wideband frequency response from 50 GHz to 300 GHz. It can be observed that the filter maintains excellent suppression across a broad stopband up to 200 GHz. A spurious passband emerges at approximately 225 GHz, which corresponds to the third harmonic of the fundamental resonance at 75 GHz. This spurious response is inherent to the quarter-wavelength resonator topology, where the third harmonic inevitably re-energizes the resonators. Figure 6b provides a detailed view of the operating band from 50 GHz to 100 GHz. Within the passband from 70 GHz to 80 GHz, the minimum insertion loss is 1.17 dB, and the return loss exceeds 18 dB. Notably, five distinct transmission zeros are visible within the passband, confirming the realization of a fifth-order Chebyshev response. Regarding out-of-band performance, the filter exhibits sharp roll-off characteristics, with rejection levels better than 60 dB at both 50 GHz and 100 GHz, demonstrating effective suppression of unwanted signals.

**Figure 7:**
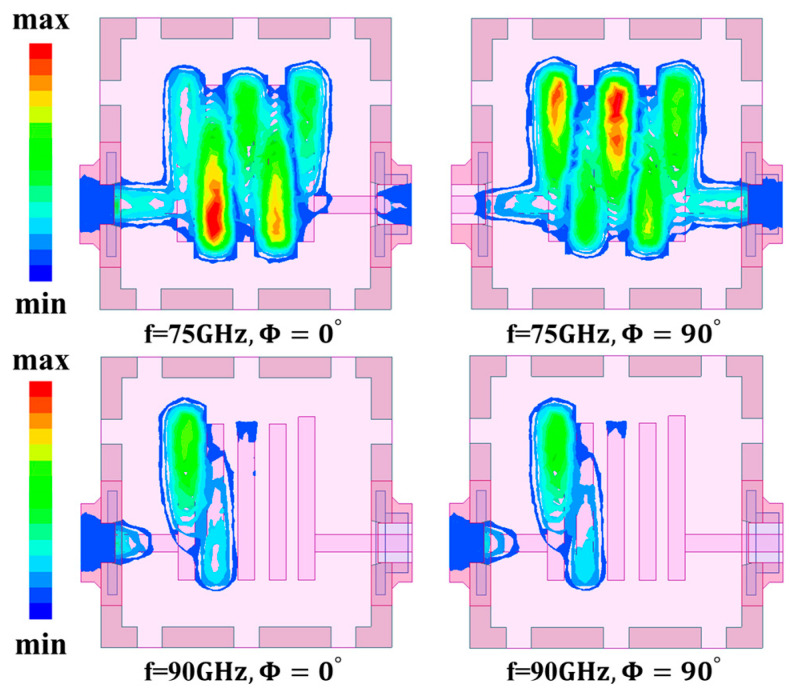
Electric field distribution at 75 GHz and 90 GHz, simulated by Ansys HFSS 2021R1.

Figure 7 illustrates the electric field distribution at 75 GHz and 90 GHz, as computed using Ansys HFSS 2021R1. The distinct patterns provide physical insight into the operating and rejection mechanisms of the proposed ARCL-based filter. Within the passband at 75 GHz, the electric field is strongly concentrated along the internal coupled transmission lines, with maximum intensity observed between the adjacent coupling sections. This confirms that the dominant mode of the filter follows a coupled-line coupling mechanism, where electromagnetic energy is efficiently transferred from the input to the output port via the parallel-coupled ARCL segments. Notably, the field remains well confined within the fully shielded air cavity, with negligible leakage into the surrounding substrate or air regions, which is a direct consequence of the enclosed metal shield and contributes to the observed low insertion loss and high selectivity. In contrast, at 90 GHz, which lies in the upper stopband, the electric field distribution exhibits a pronounced change. The field concentration along the coupled lines is substantially diminished, and instead, localized standing-wave patterns emerge with field maxima appearing at the input and output feed regions. This shift indicates that the coupled-line section no longer provides an efficient coupling path at this frequency. Consequently, energy becomes trapped at the ports rather than being transmitted across the filter, effectively creating a transmission zero in the upper stopband. Such decoupling behavior is essential for achieving wide stopband rejection, which is critical for suppressing out-of-band interference in dense spectral environments, such as those encountered in surgical and autonomous mobile robotic platforms. The final geometric parameters of the 70–80 GHz ARCL-based bandpass filter are summarized in Table 1.

## 3. Results and Discussion

**Figure 8:**
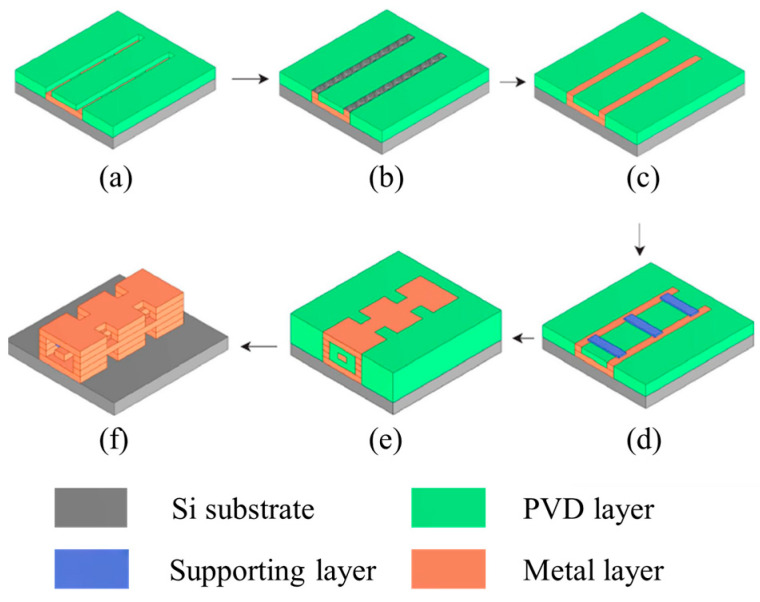
MEMS-based fabrication steps of ARCL: (**a**) lithography, (**b**) electroplating, (**c**) CMP, (**d**) spin-coating and patterning, (**e**) multi-layer stacking, and (**f**) release.

The proposed filter based on ARCL is realized on a 700 μm thick silicon wafer through standard MEMS fabrication techniques. A sequence of PVD deposition, thick photoresist lithography, electroplating, and CMP processes was used in the fabrication, as shown in Figure 8. For the 50 μm-thick single-layer electroplating process, the foundry can achieve a metal sidewall verticality better than 88° by using positive thick photoresist with optimized exposure and baking conditions. This ensures well-defined conductor profiles and reliable multilayer stacking. A metal seed layer is deposited and patterned on the substrate via PVD (a). The bottom metal structure is formed by electroplating (b). The surface is planarized by CMP to ensure a flat foundation (c). A supporting layer is defined by spin-coating and lithography to create the air cavity (d). The metal layers M2–M5 are constructed via a multi-layer stacking process (e). Finally, the sacrificial photoresist is removed by wet stripping to release the structure (f).

**Figure 9:**
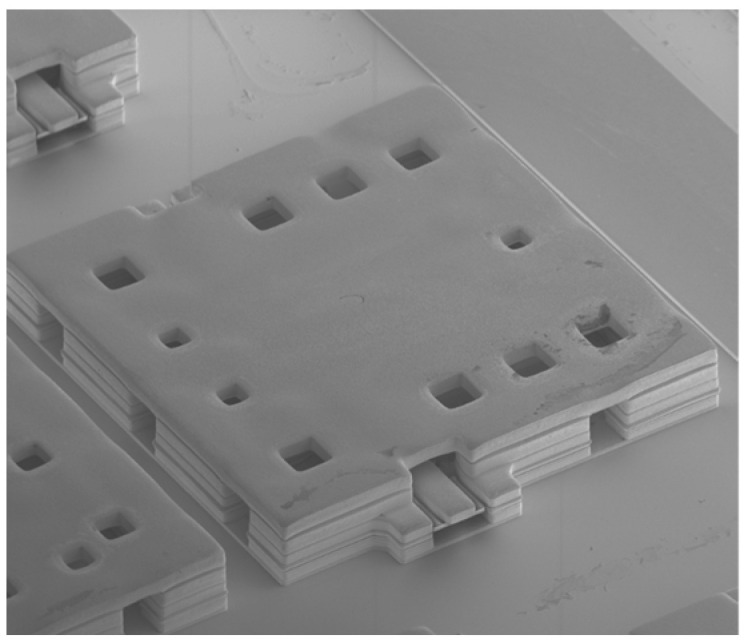
Microscopic photograph of the proposed filter.

Figure 9 presents the microscopic photograph of the proposed bandpass filter. The multilayer conductors stacked vertically on top of each other, as well as the strategically placed release holes for sacrificial layer removal, can be clearly observed. The overall chip footprint is 2000 μm×1750 μm×250 μm.

**Figure 10:**
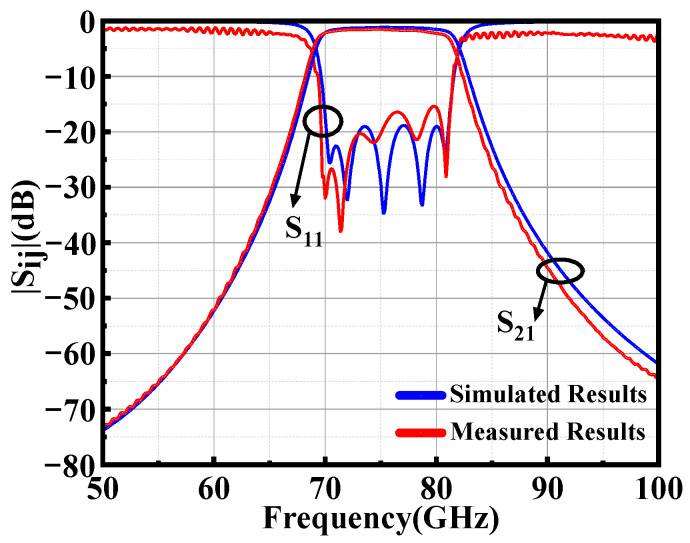
Simulated and measured results for proposed ARCL-based filter.

Figure 10 presents the simulated and measured frequency responses of the proposed filter. The measured results reveal a center frequency of 75 GHz with a 3 dB fractional bandwidth of 15.2%, and a shift of 0.95 GHz is observed between the simulated and measured results. Within the passband, five distinct resonant poles are clearly observable. The measured in-band return loss exceeds 15 dB, and the minimum insertion loss is 1.5 dB, which is slightly higher than the simulated value of 1.17 dB.

The observed discrepancies between measured and simulated results primarily stem from cumulative manufacturing inaccuracies inherent to the MEMS fabrication process. These fabrication errors include deviations in conductor linewidth and layer thickness, over-etching or under-etching, random surface roughness on metal sidewalls, and incomplete removal of sacrificial photoresist due to insufficient reaction with the etchant. Such structural imperfections alter the equivalent capacitance and inductance of the coupled-line sections, as well as the effective permittivity of the air cavity, thereby degrading the overall frequency response. Additionally, assembly-related misalignments and measurement uncertainties, such as variations in GSG probe contact position on the input/output pads, may contribute to port impedance mismatches, particularly given the high sensitivity of S-parameters at millimeter-wave frequencies.

To evaluate the performance of the proposed design, a comparative analysis with recently reported W-band bandpass filters is provided in Table 2. The GaAs-based SIW filter [25] achieves a slightly smaller on-chip footprint using ICP etching, but suffers from a relatively high insertion loss of 4.3 dB due to dielectric dissipation in the semiconductor substrate. The E-plane waveguide filter using a compact beeline resonator [26] demonstrates low insertion loss and high selectivity, yet its integration with planar front-ends is hindered by the requirement for a bulky WR-10 waveguide housing. The gap-waveguide filter employing the MEMS technique [27] features a lightweight and surface-mountable package, but its large volume limits its suitability for highly compact robotic radar modules. Compared with the ARCL-based filters in [17,28], the proposed design achieves a comparable insertion loss and a wider fractional bandwidth, while occupying a significantly smaller volume. Specifically, the third-order Chebyshev filter using open/short stubs in [17] and the fifth-order dual-behavior resonator filter using five paralleled DBRs in [28] both require larger footprints to realize their responses. In contrast, the proposed topology enables a more compact layout without sacrificing electrical performance. This compactness makes it particularly attractive for integration into autonomous robotic perception systems where payload capacity is severely constrained.

## 4. Conclusions

This article presents an ultra-compact W-band bandpass filter based on a MEMS air-cavity structure. Measurement results demonstrate that the proposed filter exhibits a miniature footprint, low insertion loss, and high selectivity. These characteristics confirm its excellent suitability and promising potential for integration into high-resolution mobile robotic radar systems and next-generation millimeter-wave sensing applications.

## Figures and Tables

**Table 1:** Geometric parameters of the 70–80 GHz MEMS cavity filter after optimization (Unit: μm).

Symbol	Value/μm	Symbol	Value/μm
$W_{m}(m=1,2\ldots 5)$	100	$s_{12},s_{45}$	73.5
$l_{n}(n=1,5)$	984	$s_{23},s_{34}$	86.5
$l_{n}(n=2,3,4)$	942	$l_{c}$	225

**Table 2:** Performance comparison of the proposed filter with state-of-the-art designs.

Ref	Form	Process	$f_{0}$ (GHz)	FBW (%)	IL (dB)	Size (mm^3^)
Prop.	ARCL	MEMS	75	1 5.2	1.5	2 × 1.75 × 0.25
[25]	SIW	GaAs ICP	93	3.4	4.3	4.2 × 2.2 × 0.07
[26]	E-Plane Waveguide	Quartz PCB	88.55	3.6	1.15	4.85 × 1.7 × 0.5
[27]	Gap W aveguide	MEMS	93.6	8	2	20 × 10 × 0.6
[17]	ARCL	Surface micromachining	94.75	7.3	1.5	3.21 × 1.27 × 0.127
[28]	ARCL	Surface micromachining	96	8	2.05	5.4 × 2.19 × 0.5

## Data Availability

Data will be made available on request.
